# Bilateral Serous Retinal Detachment in Severe Preeclampsia: A Report of Two Cases

**DOI:** 10.7759/cureus.108945

**Published:** 2026-05-16

**Authors:** Latifa Oumaiouf, Hanane Mourouth, Meryem Essafti, Houssam Rebahi, Ahmed Rhassane El Adib

**Affiliations:** 1 Anesthesia and Critical Care, Mohammed VI University Hospital, Marrakech, MAR; 2 Anesthesia and Intensive Care Unit, Mohammed VI University Hospital, Marrakech, MAR; 3 Anesthesia and Critical Care, Faculty of Medicine and Pharmacy, Cadi Ayyad University, Marrakech, MAR; 4 Anesthesia, Critical Care, and Emergency Medicine, Mohammed VI Faculty of Medicine, Mohammed VI University of Sciences and Health, Casablanca, MAR

**Keywords:** hellp syndrome, obstetric complications, preeclampsia, retinal detachment, serous retinal detachment, visual loss

## Abstract

Serous retinal detachment (SRD) is an uncommon but vision-threatening manifestation of hypertensive disorders of pregnancy, particularly in the context of severe preeclampsia and hemolysis, elevated liver enzymes, and low platelet count (HELLP) syndrome. We report two cases of young primigravid women who developed bilateral SRD associated with HELLP syndrome. Both patients presented with acute bilateral vision loss and were diagnosed using optical coherence tomography. Management included antihypertensive therapy, magnesium sulfate, and delivery without any ophthalmological intervention. Both patients experienced complete visual recovery within two weeks. This report underscores the importance of prompt recognition, appropriate obstetric management, and the typically favorable visual prognosis of SRD in preeclampsia.

## Introduction

Hypertensive disorders of pregnancy, including preeclampsia and hemolysis, elevated liver enzymes, and low platelet count (HELLP) syndrome, represent a major cause of maternal and perinatal morbidity and mortality worldwide. Preeclampsia complicates approximately 2-8% of pregnancies globally [1], while HELLP syndrome occurs in 0.5-0.9% of all pregnancies and in up to 20% of cases with severe preeclampsia [2]. Visual disturbances have been reported in up to 25% of women with severe preeclampsia, ranging from blurred vision and photopsia to cortical blindness [3,4], though the true prevalence of structural complications such as serous retinal detachment (SRD) remains incompletely defined.

The pathophysiology is rooted in systemic endothelial dysfunction and dysregulation of angiogenic factors, most notably an imbalance between soluble fms-like tyrosine kinase-1 (sFlt-1) and placental growth factor (PlGF) [5], which drives choroidal ischemia and hyperpermeability. This disrupts the retinal pigment epithelium's (RPE) fluid-pumping function, allowing subretinal fluid to accumulate beneath the neurosensory retina, producing SRD [6,7]. Because the macula lies within this vulnerable zone, even shallow detachments can cause rapid, potentially reversible central vision loss, making early ophthalmic evaluation critical [8-10].

Severe preeclampsia can also manifest through life-threatening neurological and hepatic emergencies. Posterior reversible encephalopathy syndrome (PRES), resulting from loss of cerebrovascular autoregulation, may present with seizures, visual disturbances, and altered consciousness, and can emerge as late as the second postpartum day [11]. HELLP syndrome additionally overlaps clinically with acute fatty liver of pregnancy (AFLP), and distinguishing these entities, which share hepatic dysfunction, coagulopathy, and thrombocytopenia, is essential to avoid misclassification and delayed intervention [12].

SRD in the context of preeclampsia or HELLP syndrome typically presents in the third trimester or early postpartum period [3,4,8], and prognosis is generally excellent with prompt conservative management. This report presents two instructive cases illustrating distinct clinical contexts within this spectrum, demonstrating the value of early ophthalmic evaluation and the potential for complete visual recovery when the underlying systemic disorder is effectively controlled [6,9,10].

## Case presentation

Case 1

A 19-year-old primigravida with no significant past medical history underwent an uncomplicated vaginal delivery at a primary care facility at term, giving birth to a neonate weighing 2700 g, with APGAR scores of 10/10 at both one and five minutes. Immediately postpartum, she developed a generalized tonic-clonic seizure lasting approximately two minutes, which resolved spontaneously. She was transferred emergently to our tertiary center. On admission, blood pressure was 180/110 mmHg. She reported a two-day antecedent history of frontal headache and tinnitus, followed by the acute onset of bilateral ocular pain and complete bilateral vision loss on the day of delivery.

**Figure 1:**
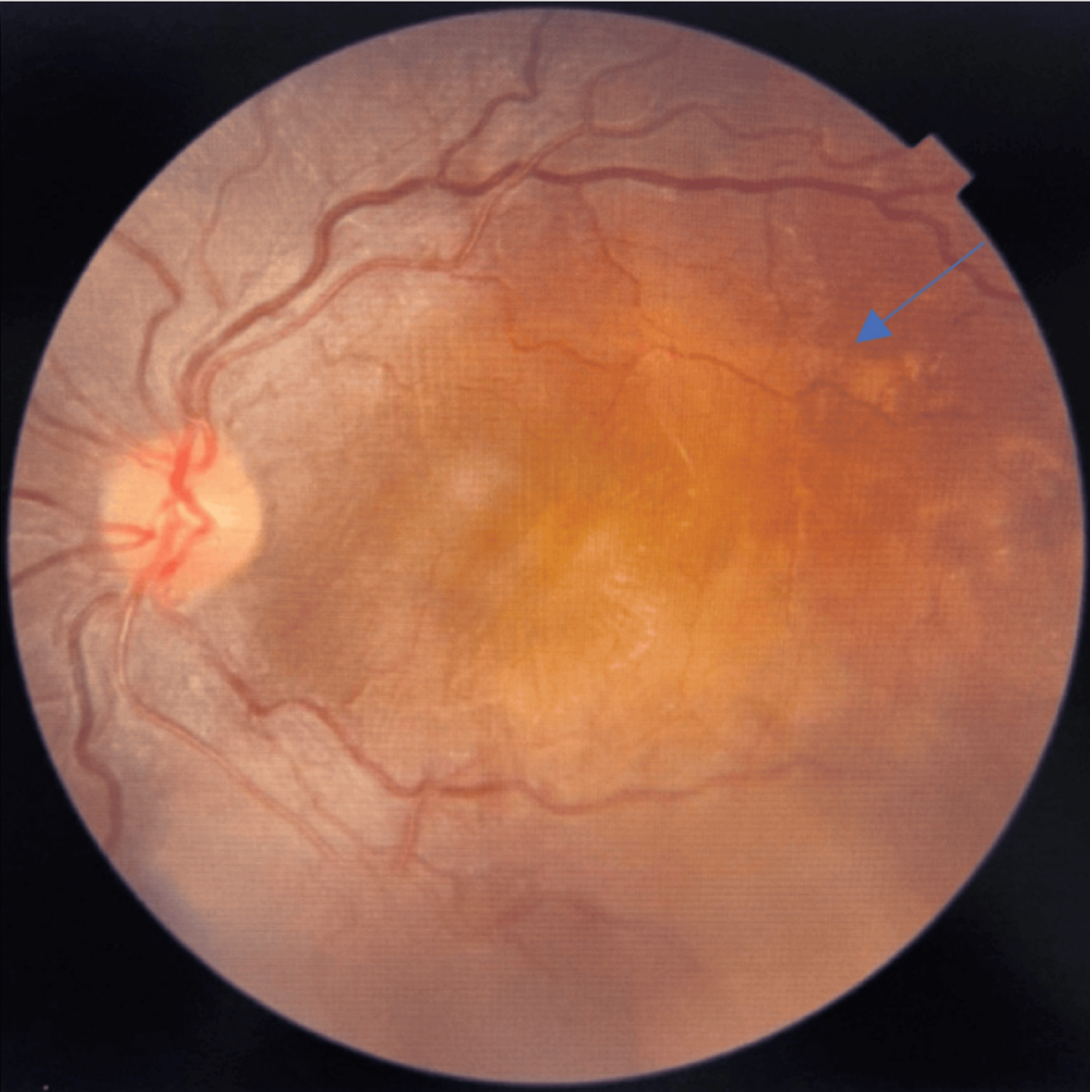
Fundus photograph of the right eye (OD) of Case 1 showing serous retinal detachment (arrow) with blurred macular reflex.

**Figure 2:**
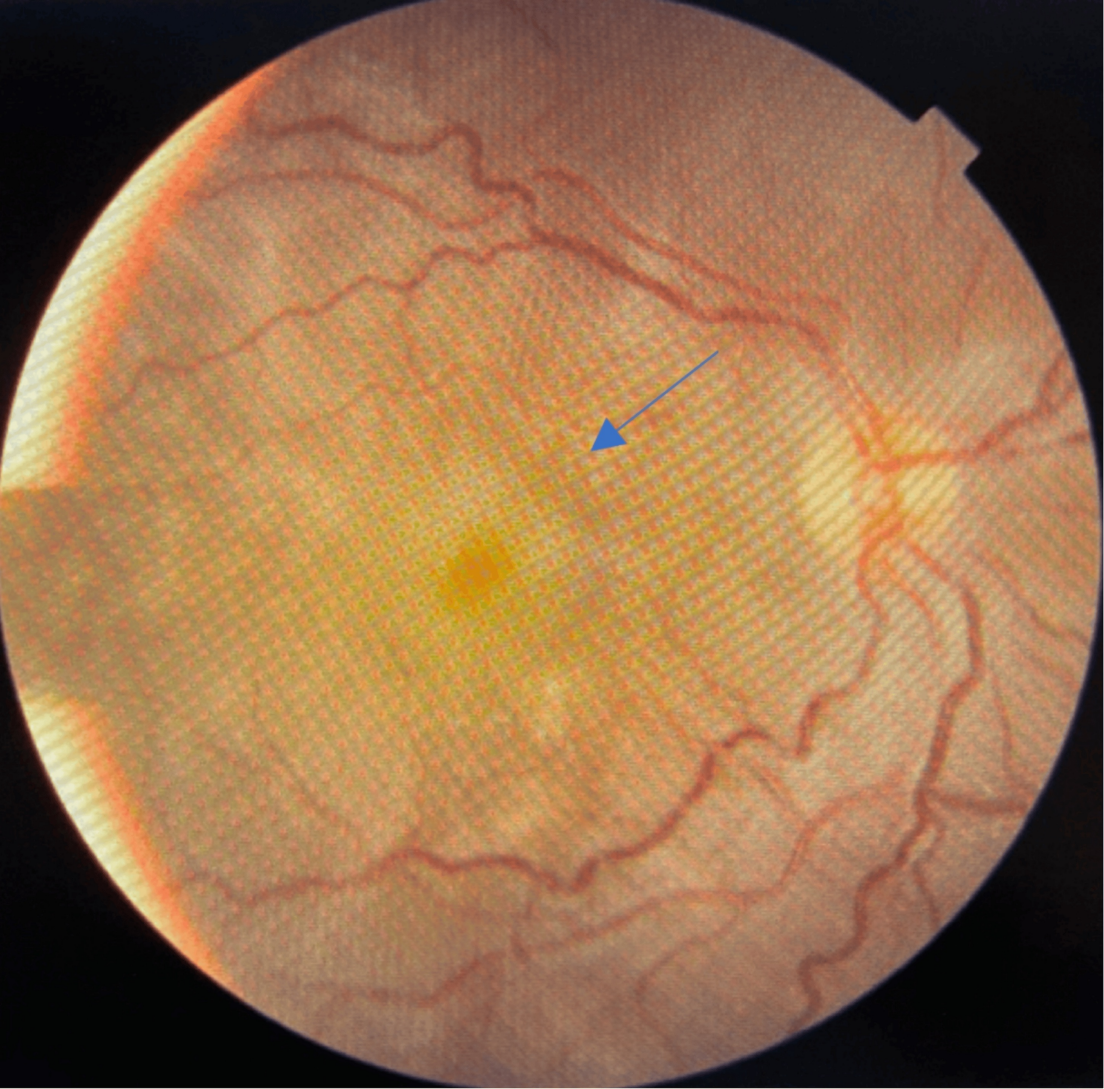
Fundus photograph of the left eye (OS) of Case 1 showing serous retinal detachment (arrow) with blurred macular reflex, similar to the right eye.

**Figure 3:**
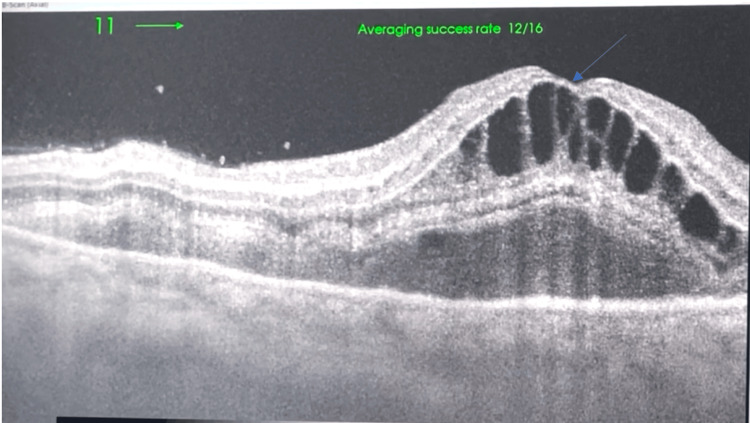
OCT of the right eye (OD) in Case 1 showing large bullous neurosensory retinal detachment with multilobular subretinal fluid accumulation (arrow).

On ophthalmological examination at admission, best-corrected visual acuity (BCVA) was hand motion (HM) in the right eye and counting fingers at 1 meter (CF/1m) in the left eye. Fundoscopy revealed bilateral SRD with bullous elevation of the posterior pole in both eyes (Figures 1-2). Optical coherence tomography (OCT) confirmed extensive subretinal fluid accumulation with neurosensory retinal detachment bilaterally, most prominent in the macular region (Figure 3). No retinal break, choroidal mass, or disc edema was identified.

Laboratory findings were consistent with HELLP syndrome: hemolysis on peripheral smear, elevated aspartate aminotransferase (AST) and alanine aminotransferase (ALT), platelet count below 100,000/μL, and heavy proteinuria at 15 g/24h (Table 1). D-dimer (DD) and soluble fibrin monomer complex (SFMC) were measured on admission as part of the venous thromboembolism (VTE) risk stratification protocol (Table 1).

**Table 1:** Comparative biological profile of the two cases at admission. g/dL: grams per deciliter; G/L: giga per liter; mg/L: milligrams per liter; IU/L: international units per liter; g/24h: grams per 24 hours; LDH: lactate dehydrogenase; AST: aspartate transaminase; ALT: alanine transaminase; *N: times the normal value

Case	Hemoglobin (g/dL)	Platelets (G/L)	Serum creatinine (mg/L)	LDH (IU/L)	AST (*N)	ALT (*N)	Uric acid (mg/L)	Proteinuria (g/24h)
Case 1	6.3	71	6.7	1845	7	11	115	15
Case 2	7.1	53	8.9	2431	10	15	143	9.4
Reference value	11.5-13	100-400	6.5-11.5	200-450	<35	<35	<70	<0.3

Management and Clinical Course

Intravenous magnesium sulfate was administered as a 4 g loading dose over 20 minutes, followed by a maintenance infusion of 1 g/hour for 24 hours. Antihypertensive therapy was initiated with intravenous nicardipine via electronic syringe infusion pump, starting at 1 mg/hour and titrated upward by 0.5 mg/hour every 15 minutes to a maximum of 5 mg/hour, targeting systolic blood pressure between 140-150 mmHg and diastolic pressure between 90-100 mmHg. Blood pressure evolved as follows: 180/110 mmHg on admission → 158/100 mmHg at six hours → 145/92 mmHg at 12 hours → 138/88 mmHg at 24 hours → 130/82 mmHg at 48 hours. Oral methyldopa (750 mg three times daily) was subsequently introduced upon weaning of intravenous therapy. Prophylactic low-molecular-weight heparin was initiated postpartum in accordance with institutional VTE prophylaxis guidelines. No ophthalmic surgical intervention was performed.

Visual recovery closely followed blood pressure normalization and subretinal fluid resorption. At day 5 postpartum, BCVA was counting fingers at 2 meters (CF/2 m) in the right eye and 20/150 in the left eye. At day 7, BCVA improved to 20/80 in the right eye and 20/60 in the left eye. At two weeks, BCVA reached 20/25 bilaterally, with OCT confirming a marked reduction of subretinal fluid. At the six-week follow-up, BCVA was 20/20 in both eyes, and OCT confirmed complete anatomical recovery with restoration of normal foveal contour.

Materno-Neonatal Outcomes

The mother was discharged on day 7 postpartum in stable condition with normalized blood pressure on oral antihypertensive therapy. No neurological sequelae were observed at discharge. Subsequent neonatal assessment by the attending pediatrician revealed no abnormal findings, and the newborn was deemed clinically stable and in good general condition.

Case 2

A 20-year-old primigravida presented at 34 weeks of gestation with a two-week history of progressive bilateral visual blurring, initially mild and intermittent, then worsening to near-complete visual impairment over the final three days before admission. She had been followed for gestational hypertension in a peripheral clinic and was referred to our facility upon documentation of severe hypertension. On admission, blood pressure was 172/108 mmHg. She denied headache or epigastric pain at presentation.

**Figure 4:**
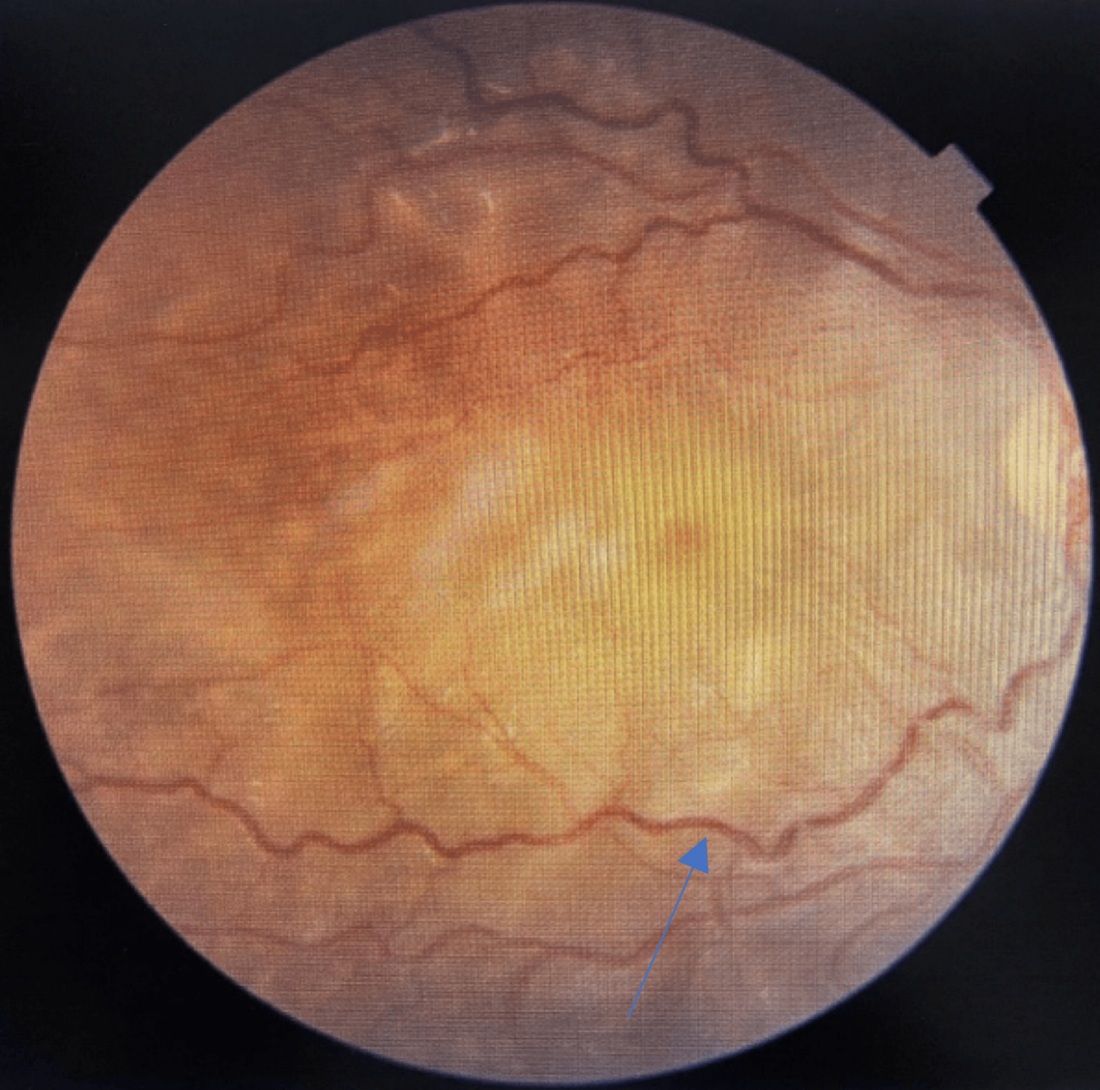
Fundus of the right eye (OD) in Case 2 showing neurosensory retinal detachment with subretinal fluid (arrow).

**Figure 5:**
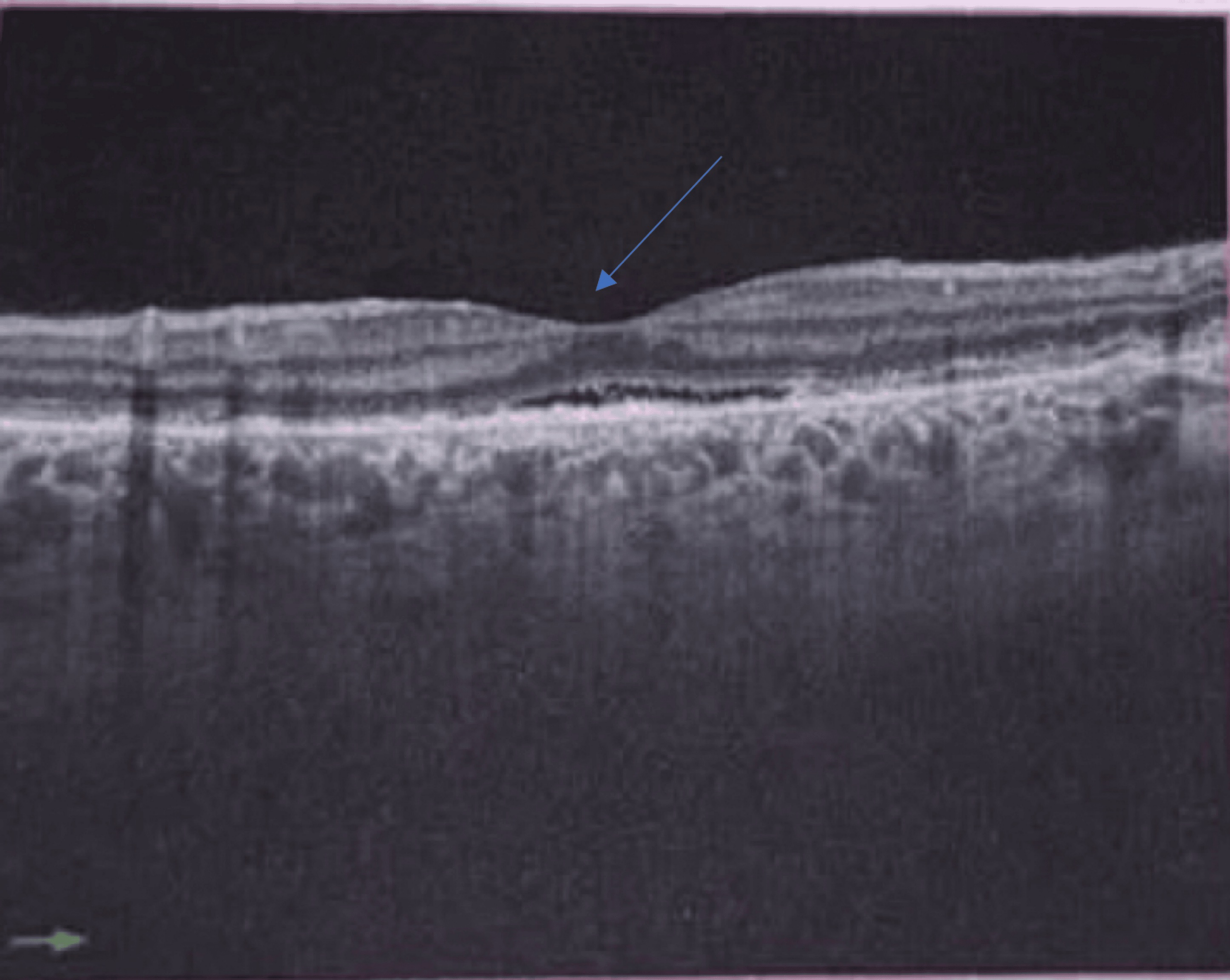
OCT of the right eye (OD) in Case 2 showing shallow neurosensory retinal detachment with subretinal fluid (arrow).

On ophthalmological examination at admission, BCVA was 20/200 in the right eye and CF/2m in the left eye. Fundoscopy revealed bilateral SRD with inferiorly shifting subretinal fluid in both eyes (Figure 4). OCT confirmed extensive bilateral subretinal fluid accumulation with macular involvement (Figure 5). No retinal break, choroidal neovascularization, or vitreous hemorrhage was identified.

Laboratory evaluation confirmed HELLP syndrome (Table 1). Given the gestational age of 34 weeks and the severity of the maternal condition, labor was induced emergently. A live neonate was delivered.

Management and Clinical Course

Intravenous magnesium sulfate was administered as a 4 g loading dose followed by 1 g/hour for 24 hours. Antihypertensive therapy was initiated with intravenous nicardipine via electronic syringe infusion pump at 1 mg/hour, titrated to a maximum of 4 mg/hour, achieving target blood pressure control within six hours of admission: 172/108 mmHg on admission → 144/92 mmHg at six hours → 132/84 mmHg at 24 hours → 128/80 mmHg at 48 hours. Oral methyldopa (750 mg three times daily) and oral nicardipine (50 mg twice daily) were introduced at 48 hours as IV therapy was weaned. Prophylactic low-molecular-weight heparin was initiated postpartum. No ophthalmic surgical intervention was undertaken.

Visual recovery closely followed blood pressure normalization and subretinal fluid resorption. At day 5 postpartum, BCVA was 20/100 in the right eye and 20/80 in the left eye. At two weeks, BCVA improved to 20/30 bilaterally, with OCT showing a marked reduction of subretinal fluid. At the six-week follow-up, BCVA was 20/20 in both eyes, and OCT confirmed complete anatomical recovery.

Materno-Neonatal Outcomes

The mother was discharged on day 8 postpartum in good clinical condition with blood pressure controlled on oral therapy. The neonate was born at 34 weeks with a birth weight of 1900 grams and APGAR scores of 10/10 and 10/10 at one and five minutes. Given prematurity, the neonate was admitted to the neonatal intensive care unit (NICU) for two days and discharged without complications.

## Discussion

SRD is a rare but significant complication of hypertensive disorders of pregnancy, especially preeclampsia and HELLP syndrome. Although it is estimated to occur in less than 1% of preeclamptic patients, the true incidence is likely underestimated due to the absence of routine ophthalmological screening in this population and the possibility of extra-macular locations, which may render the condition clinically silent [3]. SRD typically presents in the third trimester or early postpartum period, and the majority of published cases describe a bilateral presentation [4]. The condition is more frequently observed in primigravidas, a well-established demographic risk factor for preeclampsia [1]. Additionally, the presence of HELLP syndrome appears to significantly amplify both the risk and severity of SRD, likely through the additive effects of hemolysis, hepatic dysfunction, and thrombocytopenia on the already compromised endothelial milieu [2].

The pathophysiology is multifactorial. Placental hypoperfusion leads to the release of anti-angiogenic factors such as sFLT-1, which binds and neutralizes vascular endothelial growth factor (VEGF) and PlGF, resulting in systemic endothelial dysfunction. This contributes to increased choroidal vascular permeability, choroidal ischemia, and disruption of the outer blood-retinal barrier at the level of the RPE [5]. Failure of the RPE's active fluid transport function allows subretinal fluid to accumulate beneath the neurosensory retina, producing the characteristic serous detachment. Fluorescein angiography in reported cases demonstrates delayed choroidal filling followed by dye leakage at the RPE level, while retinal vessels are usually unaffected - a pattern that helps distinguish this entity from primary hypertensive retinopathy with arteriolar changes [6]. Indocyanine green angiography further confirms choroidal hyperpermeability and lobular ischemia [7]. OCT findings typically reveal subretinal fluid accumulation beneath the neurosensory retina and, in some cases, associated RPE detachment [4,6]. Serial OCT imaging provides objective, quantitative documentation of subretinal fluid resolution and foveal anatomy recovery, enabling precise correlation between systemic blood pressure control and retinal improvement over the postpartum course.

Beyond direct ophthalmoscopic and OCT evaluation, ophthalmic artery Doppler ultrasound has emerged as a promising non-invasive adjunct in the hemodynamic assessment of severe preeclampsia. As the first intracranial branch of the internal carotid artery, the ophthalmic artery provides a sonographically accessible window into cerebrovascular resistance and intracranial pressure dynamics. Elevated ophthalmic artery pulsatility index and reversal of the normal flow pattern have been associated with impaired cerebrovascular autoregulation and increased risk of neurological complications, including PRES, a severe peripartum complication that may manifest as late as the second postpartum day with seizures, visual disturbances, and altered consciousness, as illustrated by Vuong et al. [11]. Its prospective integration into the multimodal monitoring protocol for women with severe preeclampsia and visual symptoms warrants systematic investigation.

Several conditions must be considered in the differential diagnosis of bilateral exudative retinal detachment in the pregnant or postpartum patient. Central serous chorioretinopathy (CSCR) typically presents as a unilateral, self-limited detachment in younger patients, often associated with stress or corticosteroid exposure, and is not accompanied by the degree of systemic hypertension or the hematological profile of HELLP syndrome. Hypertensive choroidopathy without HELLP may produce bilateral exudative detachments in the context of accelerated hypertension of any etiology, but is distinguished by the absence of hemolysis, elevated liver enzymes, and thrombocytopenia. Vogt-Koyanagi-Harada (VKH) disease can produce striking bilateral exudative detachments and must be actively excluded; it is characteristically associated with uveitis, disc hyperemia, choroidal thickening on OCT, and systemic features including meningismus, vitiligo, and alopecia. Uveal effusion syndrome and choroidal metastasis are further diagnostic considerations, distinguished respectively by their characteristic peripheral retinal and choroidal imaging features. The convergence of severe systemic hypertension, the HELLP laboratory profile, bilateral symmetrical macular detachment without retinal break or choroidal mass, and complete spontaneous resolution following blood pressure control are the key discriminating features that confirm the diagnosis of preeclampsia/HELLP-associated SRD.

A critical diagnostic pitfall in patients presenting with HELLP syndrome is its clinical overlap with AFLP, a distinct but equally life-threatening obstetric emergency. Both conditions share features of hepatic dysfunction, coagulopathy, and thrombocytopenia, and misclassification may delay life-saving interventions. AFLP is characteristically associated with microvesicular hepatic steatosis, hypoglycemia, elevated ammonia, and progressive hepatic encephalopathy - features that help distinguish it from HELLP syndrome. As highlighted by Nguyen and Ho [12] in a detailed case report and literature review, the distinction between these two entities demands careful integration of clinical, biochemical, imaging, and, when necessary, histopathological data.

HELLP syndrome is also associated with significant activation of the coagulation cascade and an elevated risk of VTE, which can further compromise maternal outcomes if unrecognized. Systematic measurement of DD and SFMC is therefore warranted as part of the peripartum laboratory workup in these patients. As demonstrated by Vuong et al. [11] in a prospective observational study of 100 third-trimester Vietnamese pregnancies stratified according to the Royal College of Obstetricians and Gynaecologists (RCOG) VTE risk score, elevated DD and SFMC concentrations are independently associated with high VTE risk in the peripartum period and provide additive discriminatory value beyond clinical scoring alone. Both patients in the present report received prophylactic low-molecular-weight heparin postpartum in accordance with institutional guidelines, with no thromboembolic events documented during follow-up.

Clinically, patients may present with blurred vision, scotomas, or complete bilateral vision loss. Visual acuity at presentation can range from mild impairment to HM perception, and its trajectory of recovery closely mirrors the normalization of systemic blood pressure and the resorption of subretinal fluid on serial OCT imaging. Despite the often dramatic onset, the prognosis is generally favorable. Spontaneous resolution of SRD within 2 to 12 weeks postpartum is consistently reported in the literature, with recovery of baseline visual acuity in the majority of patients [2,4,8]. The speed and efficacy of blood pressure normalization appear to be the most important modifiable prognostic variable, with early and sustained antihypertensive control associated with faster visual recovery. Importantly, no specific ocular treatment is required; conservative systemic management of preeclampsia or HELLP syndrome - comprising intravenous antihypertensive therapy, seizure prophylaxis with magnesium sulfate, and timely delivery - is sufficient to achieve full visual and anatomical recovery [2,8].

Interestingly, a few rare cases of SRD have been reported even in the absence of significant hypertension, underscoring the independent contribution of endothelial dysfunction and choroidal vascular dysregulation beyond blood pressure elevation alone [9]. This observation reinforces the importance of a broad clinical approach: any pregnant or postpartum patient presenting with visual disturbances should undergo prompt ophthalmological evaluation to rule out SRD and guide obstetric decision-making, regardless of the degree of blood pressure elevation. Conversely, the incidental detection of SRD in a pregnant woman should prompt urgent investigation for underlying hypertensive disorders of pregnancy, even in the absence of overt systemic symptoms [10].

SRD associated with preeclampsia and HELLP syndrome is a rare but important diagnosis requiring a high index of clinical suspicion and a coordinated multidisciplinary approach involving obstetricians, ophthalmologists, and intensivists. Although the clinical presentation can be alarming, the prognosis is generally excellent with prompt conservative management. Systematic ophthalmological evaluation, serial OCT monitoring, structured antihypertensive titration, VTE risk stratification, and careful exclusion of diagnostic mimics - including AFLP and PRES - are essential components of optimal care. Prompt recognition and appropriate obstetric management are essential to ensure maternal and fetal safety while preserving visual function.

## Conclusions

SRD remains a rare but clinically significant ocular manifestation of hypertensive disorders of pregnancy, particularly severe preeclampsia and HELLP syndrome. The two cases presented in this report add to the growing body of literature supporting the generally favorable visual prognosis of this condition when systemic stabilization is achieved promptly through antihypertensive therapy, seizure prophylaxis, and timely delivery. Complete anatomical and functional recovery, confirmed by serial OCT imaging and visual acuity assessment, was achieved in both patients without specific ocular intervention. However, given the inherent limitations of a two-case report, broader generalizations regarding incidence, standardized management protocols, or definitive prognostic indicators should be drawn with caution. The observations presented here are hypothesis-generating rather than conclusive, and prospective multicenter studies with larger patient cohorts are needed to better define the true incidence of SRD in the context of hypertensive disorders of pregnancy, identify reliable prognostic markers, and establish evidence-based guidelines for systematic ophthalmic screening and follow-up in this population.

Early recognition of visual disturbances in pregnant or postpartum women with hypertensive disorders remains essential, and prompt ophthalmological evaluation should be considered a standard component of multidisciplinary care in this setting. Closer collaboration between obstetricians, ophthalmologists, and intensivists is key to ensuring optimal maternal and neonatal outcomes while preserving visual function.

## References

[1] Duckitt K, Harrington D (2005). Risk factors for pre-eclampsia at antenatal booking: systematic review of controlled studies. BMJ.

[2] Teodoru CA, Tudor C, Cerghedean-Florea ME (2023). Bilateral serous retinal detachment as a complication of HELLP syndrome. Diagnostics (Basel).

[3] Raposo JT, Melo BC, Maciel NF, Leite SD, Rebelo ÓRC, Lima AM (2020). Serous retinal detachment in pre-eclampsia: case report and literature review. Rev Bras Ginecol Obstet.

[4] Benlghazi A, Bouhtouri Y, Belouad M (2023). Bilateral serous retinal detachment in pre-eclampsia a rare but favorable complication: case report. Oxf Med Case Reports.

[5] Tsatsaris V, Fournier T, Winer N (2008). Pathophysiology of preeclampsia [Article in French]. J Gynecol Obstet Biol Reprod.

[6] Sathish S, Arnold JJ (2000). Bilateral choroidal ischaemia and serous retinal detachment in pre-eclampsia. Clin Exp Ophthalmol.

[7] Valluri S, Adelberg DA, Curtis RS, Olk RJ (1996). Diagnostic indocyanine green angiography in preeclampsia. Am J Ophthalmol.

[8] Jayaraj S, Samanta R, Puthalath AS, Subramanian K (2020). Pre-eclampsia associated bilateral serous retinal detachment. BMJ Case Rep.

[9] Santoro JM, Jeng-Miller KW, Liang MC (2023). Bilateral serous retinal detachments in a patient with preeclampsia without hypertension. Retin Cases Brief Rep.

[10] Gupta R, Sheidow T (2019). Bilateral serous retinal detachment in association with preeclampsia. Can J Ophthalmol.

[11] Vuong AD, Pham XT, Nguyen PN (2024). Posterior reversible encephalopathy syndrome (PRES) on the second postpartum day: learning experience from a case report and literature review. Int J Emerg Med.

[12] Nguyen PN, Ho QN (2025). Acute fatty liver disease in full-term pregnancy: a case report and review of the literature. J Med Case Rep.

